# Positive Effect of Andrographolide Induced Autophagy on Random-Pattern Skin Flaps Survival

**DOI:** 10.3389/fphar.2021.653035

**Published:** 2021-03-16

**Authors:** Jingtao Jiang, Jie Jin, Junsheng Lou, Jiafeng Li, Hongqiang Wu, Sheng Cheng, Chengji Dong, Hongyu Chen, Weiyang Gao

**Affiliations:** ^1^ Department of Orthopaedics, The Second Affiliated Hospital and Yuying Children’s Hospital of Wenzhou Medical University, Wenzhou, China; ^2^ Zhejiang Provincial Key Laboratory of Orthopaedics, Wenzhou, China; ^3^ The Second Clinical Medical College of Wenzhou Medical University, Wenzhou, China

**Keywords:** random-pattern flap, andrographolide, autophagy, angiogenesis, apoptosis, PI3K/Akt signaling pathway, oxidative stress

## Abstract

Random-pattern skin flap replantation is generally used in the reconstruction of surgical tissues and covering a series of skin flap defects. However, ischemia often occurs at the flap distal parts, which lead to flap necrosis. Previous studies have shown that andrographolide (Andro) protects against ischemic cardiovascular diseases, but little is known about the effect of Andro on flap viability. Thus, our study aimed to building a model of random-pattern skin flap to understand the mechanism of Andro-induced effects on flap survival. In this study, fifty-four mice were randomly categorized into the control, Andro group, and the Andro+3-methyladenine group. The skin flap samples were obtained on postoperative day 7. Subsequently, the tissue samples were underwent a series of evaluations such as changes in the appearance of flap tissue, the intensity of blood flow, and neovascularization density of skin flap. In our study, the results revealed that Andro enhanced the viability of random skin flaps by enhancing angiogenesis, inhibiting apoptosis, and reducing oxidative stress. Furthermore, our results have also demonstrated that the administration of Andro caused an elevation in the autophagy, and these remarkable impact of Andro were reversed by 3-methyladenine (3-MA), the most common autophagy inhibitor. Together, our data proves novel evidence that Andro is a potent modulator of autophagy capable of significantly increasing random-pattern skin flap survival.

## Introduction

The random-pattern skin flap is commonly used in the reconstruction of defective skin tissues because it is a simple, reliable, and convenient tool (Chehelcheraghi et al., 2016; Fichter et al., 2016; Pu et al., 2017). However, flap necrosis is one of the most common complication after surgery due to inadequate blood flows, especially the length-to-width ratio of flaps exceeds 2:1 (Chehelcheraghi et al., 2016; Lin et al., 2018). The blood supply of the skin flap mainly depends on the network of the microvascular flap pedicle and the angiogenesis starting from the pedicled flap and extending to the distal part (Lin et al., 2017; Wang et al., 2017). Subsequently, the blood flow at the distal part is usually poor and insufficient, leading to ischemic necrosis (Li et al., 2019). Moreover, after neovascularization, the recovery and blood flow reperfusion cause ischemia-reperfusion injury (IRI) of ischemic tissues (Siemionow and Arslan, 2004). Random-pattern skin flap often exists local hypoxia in its distal tissue under hypoperfusion (Sies et al., 2017). During this process, the production of reactive oxygen species (ROS) continues to increase until the intracellular environment is out of balance, and a series of oxidation reactions are responsible for variations in cellular structures and their functions, ultimately results in tissue injury (Wu and Bratton, 2013). Various published studies demonstrated that the IRI induces ROS accumulation and apoptosis of functional cells, which ultimately contribute to necrosis of skin flap (Gurlek et al., 2006; van den Heuvel et al., 2009; Ren et al., 2018). Considering these mechanisms, the potential treatments can start with promoting angiogenesis, reducing oxidative stress and apoptosis.

Andrographolide (Andro), a bioactive constituent of the Chinese medicinal plant Andrographis paniculata. Andro is commonly used to fighting against the inflammatory and apoptotic processes. However, recent research reveals that Andro had benefical effects in other systems and organs. Furthermore, Andro also regulated HG (high glucose)-induced damage through activation of PI3K/AKT-eNOS signaling cascade in human umbilical vein endothelial cells, thereby promoting blood vessel formation (Duan et al., 2019). It attenuated depression-like behavior caused by chronic unpredictable mild stress through triggering autophagy-mediated inflammation inhibition (Geng et al., 2019). However, there still remains unclear that the effect of Andro and Andro-induced autophagy after skin flap reconstruction.

Autophagy is a very conserved process that degrades the accumulation of toxic and necrotic substances and maintains the stability of the intracellular environment (Nath et al., 2020). Autophagy has anti-aging and anti-tumorigenic effects and inhibits neurodegeneration, degrades invading microbes, and presents intracellular antigens. So, autophagy is crucial for eukaryotic cells survival under harsh environments (Itakura and Mizushima, 2010). Nowadays, researchers pay more attention to autophagy that contributes significantly to many diseases. However, autophagy does not show only a positive side during disease progression, its excessive activation may also result in cell death during acute myocardial infarction (Zou et al., 2019). Earlier findings have proved that autophagy plays a specific role at a certain time. Therefore, this study is aimed to exploring the effect of Andro and Andro-induced autophagy on the random skin flap survival.

## Materials and Methods

### Animals

Fifty-four C57BL/6 mice (2 months old male, 20–30 g) were procured from the laboratory animal center of Wenzhou Medical University (license no. SYXK [ZJ] 2020-0014) and were individually retained in a place which was maintained according to the standard recommendations for animal housing such as humidity (60–70%) and temperature (22–25°C). Animal treatment and care were strictly followed by the recommended suggestions on animal experimentation of Laboratory Animals of the China National Institutes of Health. The approval for these experimental methods was provided via the Animal Research Committee of Wenzhou Medical University (wydw2017-0022). All animals were treated with extreme care in order to decrease the pain of animals. In this study, mice breading were carried out separately in a maintained cage with standard experimental conditions such as 12 h light and 12 dark cycles (1:1). Food and water were provided all-time with easy access. The mice were classified (in a randomized manner) as andrographolide, Andrographolide+3-methyladenine (3MA), and control group. Each group comprised of 18 mice (n = 18).

### Antibodies and Reagents

**FIGURE 1:**
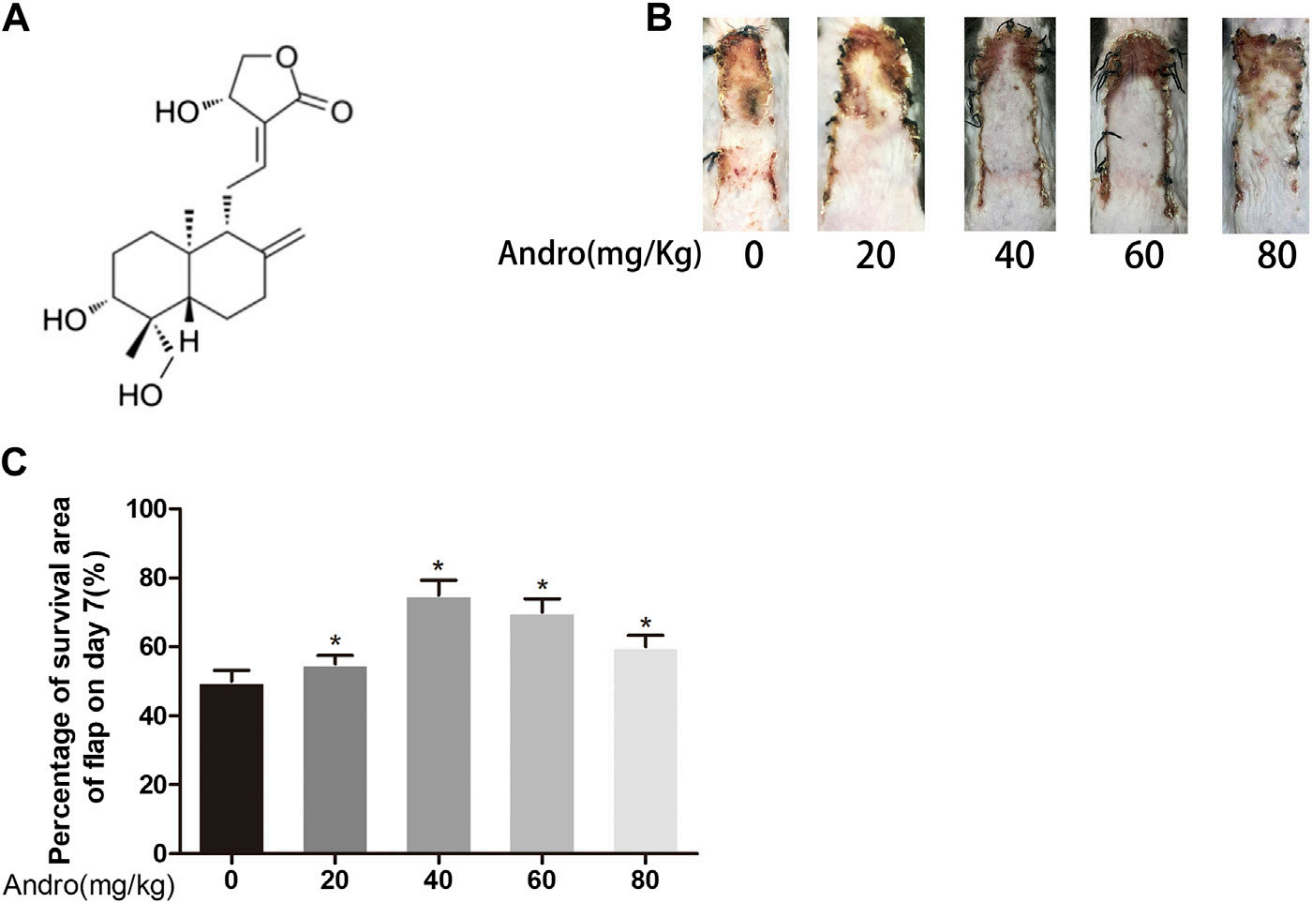
Dose-dependent effect of Andro on random pattern flap survival **(A)** Chemical structure of Andrographolide. **(B)** Effects of different concentrations of Andrographolide on the survival/necrosis area after operation (7th day). **(C)** The histogram of survival area percentage on postoperative day seventh. The obtained data were presented as means ± SEM. Significance: *p-value < 0.05 and **p-value < 0.01, vs. control group (n = 6 per group).

Andrographolide (C_20_H_30_O_5_, purity ≥97.46%; Figure 1A) was procured from a Chinese company i.e., MedChemExpress, Shanghai. Anti-GAPDH rabbit monoclonal antibodies were provided by Biogot Technology (AP0063; Shanghai, China). The rabbit monoclonal anti-VPS34 (12452-1), anti-VEGF (1003-1), anti-SOD1 (10269-1), anti-MMP9 (10375-2), anti-CAPS3 (19677-1), anti-CTSD (21327-1) and anti-HO1 (10701-1) were procured from Chicago, IL, United States. Monoclonal anti-eNOS antibody (rabbit, 12994), anti-Bax (32027), and anti-cytochrome *c* (CYC, 14796) were provided by Cell Signaling Technology, United Staes. Monoclonal anti-SQSTM1/p62 of the mouse (ab56416) was acquired from Abcam, Cambridge, United Kingdom. Monoclonal anti-LC3 of rabbit (L7543) and 3MA (M9281) were procured from Sigma-Aldrich. HRP conjugated IgG Secondary antibodies were acquired from Santa Cruz Biotechnology. FITC conjugated IgG secondary antibodies were acquired from a Chinese company Boyun Biotechnology. The Electrochemiluminescence (ECL) Plus Reagent Kits were provided by PerkinElmer Life Sciences (Massachusetts, United States). The BCA kits were procured from ThermoFisher Scientific, United States while the DAPI (4′, 6-diamidino- 2-phenylindole) solution was procured from Beyotime Biotechnology (Jiangsu, China). Diaminobenzidine (DAB) developer, pentobarbital sodium, and the H&E Kit were procured from Solarbio Science and Technology (Beijing, China), while Cdh5-Rabbit Monoclonal Antibodies were provided via Boster Biological Technology, China.

### Flap Animal Model

All animals were sedated via 1% (w/v) pentobarbital sodium (50 mg/kg) injections intraperitoneally. Next, according to the earlier reported procedure (Lee et al., 2017), a 1.5/4.5 cm caudally-based random-pattern flap elevation was carried out in the mouse dorsum, underneath the panniculus carnosus. Each supporting vessel of the flap was torn apart. Lastly, the divided flaps were instantly sutured to the donor bed via non-absorbable silk (4-0). Each flap was demarcated into three separate equal zones: area I (proximal with the caudal base of the flap), area II (intermediate), area III (distal zones). On postoperative day 7, the mice were euthanized via pentobarbital sodium overdosing. One-third of mice per group were used for immunoblotting and the other one-third of mice per group were used for IHC immunofluorescence and H&E stain. The remaining one-third of mice were used to evaluate the flap viability, tissue edema, laser Doppler blood flow (LDBF), and digital photography after surgery.

### Drug Administration

Two months old male C57BL/6 mice (20–30°G) were distributed in three separate groups (eighteen mice per group). Andrographolide was solubilized in 0.9% saline and 2% DMSO to prepare a 50 mg/ml andrographolide solution as reported reviously (Alawi et al., 2015). To investigate the dose-response to Andrographolide *in vivo* experiments, different concertrations of Andro (0, 20, 40, 60, 80 mg/kg) were injected to the mice via intraperitoneal injection (Figures 1B,C). 3MA was given to the andrographolide+3MA group (15 mg/kg) before andrographolide administration. The control group was exposed to the equivalent volume of the vehicle on daily basis. All the mice were treated with the above administration method for one week consecutively. The animals were sedated via excessive pentobarbital sodium amount, followed by harvesting the skin flap tissues and then preserved in 4% paraformaldehyde.

### Evaluation of Flaps Survival

On the third and seventh day after surgery, the random skin flaps viability was estimated via high-class photography. Macroscopic development and visualization, hair state of the flap, and color were observed on postoperative day 7 (POD 7). The surviving area was evaluated via Image-Pro Plus imaging (version 6.0), using the underlined calculation.

Percentage viable (%) = extent of viable area/whole flap.

### Appraisement of Tissue Edema

Tissue edema is considered to be a significant factor associated with ischemic flap necrosis and, therefore, the level of edema is a key indicator of the inclination of necrosis. Tissue edema was revealed based on the water content of the flaps. Six flap tissue samples per group were weighed on day seventh after surgery via dehydrating the samples for 2 days (at 50°C temperature) in order to obtained accurate weight (dry weight). The percentage of water was identified via the following calculations [(wet weight − dry weight)/wet weight] × 100%.

### Laser Doppler Blood Flow Imaging

The blood supply below the flap was evaluated via LDBF imaging. The mice were sedated and retained in a stable position on POD 7. Laser Doppler instrument, United Kingdom, was employed for the scanning of the entire dorsal skin site to evaluate microvascular blood flow. Laser Doppler provides deeper penetration, enhancing the visualization of microvessels underneath the skin surface. The obtained data were quantified via moor LDI version 6.1 software (Moor Instruments), while the intensity of blood flow was evaluated via perfusion units (PU). For each animal, equal scans were measured thricely.

### H&E Staining

After surgery for one week, six samples (1 × 1 cm) from area II were acquired in each group for histopathologic analysis. The underlined samples were stored in 4 percent paraformaldehyde for 24 h and then for transverse sectioning the samples were embedded in paraffin wax, which was divided into 4 mm in thickness for H&E staining, followed by evaluating the histological changes through light microscopy with ×200 magnification. To assess the condition of endogenous angiogenesis, MVD (microvascular density) was measured via the total number of vessels per unit area (/mm^2^) from the randomly selected part in per area II of flap tissue.

### Immunohistochemistry

In each group, the deparaffinization of the tissue sections of area II was carried out in xylene, followed by rehydration in ethanol. Hydrogen peroxide solution (3%) was added for the blockage of endogenous peroxidase activity and then repaired the antigen retrieval via sodium citrate buffer at pH 6.0 (10.2 mM) in a microwave oven. After cooling at room temperature, the slides were treated with primary antibodies i.e., Cadherin5 (1:100), CD34 (1:200), CTSD (1:100), CASP3 (1:200), SOD1 (1:100) and VEGF (1:300) at 4°C. The next specimen was handled with an HRP-conjugated secondary antibody (1:1,000), stained with a DAB detection kit, and counterstained with hematoxylin. Finally, the stained sections photograph were taken via light microscopy (×200 optical magnification) using the DP2-TWAIN image acquisition system, Japan. Image-Pro Plus was employed in order to calculate the integrated absorbance of Cadherin5-, SOD1-and CTSD-, CASP3-, VEGF-, and the CD34-positive blood vessels. In three random sections, the statistical calculation was obtained from six random visual fields.

### Immunofluorescence

Deparaffinization of embedded tissue sections was carried out in xylene, followed by rehydration in graded ethanol. Next, at 95°C, the tissue antigens were repaired via sodium citrate buffer (10.2 mM) for 20 min. The blocking was carried out via 10 percent (v/v) BSA in PBS for 1 h, followed by incubating the slides (at 4°C) with an anti-LC3II monoclonal antibody (1:200) for 24 h. Then, the tissue sections were reincubated with FITC-conjugated secondary antibody for 1 h at approximately 25°C. Cells in the dermal layer were observed using a fluorescence microscope, Japan for the evaluation of LC3II-positivity.

### Westen Blotting

For western blotting, tissue samples were collected from Area II of the flap before the mice had been euthanized. The flap of Area II per group was extracted by lysis buffer, followed by conducting the BCA assay in order to measure the protein contents. An equivalent quantity of protein was isolated via 12.5% (w/v) gel electrophoresis, followed by transferring to PVDF membranes. At approximately 25°C, the membrane blockage was performed with 5% defatted milk powder, followed by membranes incubation (at 4°C, for 24 h) with the primary antibodies i.e., MMP-9 (1:1,000), cadherin 5 (1:1,000), VEGF (1:1,000), p62 (1:1,000), HO1 (1:1,000), eNOS (1:1,000), SOD1 (1:1,000), CYC (1:1,000), caspase3 (CAPS3)(1:1,000), Beclin 1 (1:1,000), GAPDH (1:1,000), LC3 (1:500), CTSD (1:1,000), VPS34 (1:1,000) and Bax (1:1,000). Next, the membranes were washed via TBS buffer along with the Tween, followed by incubating with secondary antibody (1:5,000) for 2 h at approximately 25°C. On the membrane, the protein bands visualization was carried out by using the ECL Plus Reagent Kit. Image Lab 3.0 Bio-Rad, United States) was employed for quantification of bands intensity.

### Statistical Analyses

The statistical analysis of obtained data was carried out via SPSS version 25 software. All data have been indicated as mean ± SEM. The mean values between two groups were compared via an independent-sample *t*-test. *p*-values <0.05 were regarded as statistically significant.

## Results

### Andro Enhances the Random-Pattern Skin Flap Survival

**FIGURE 2:**
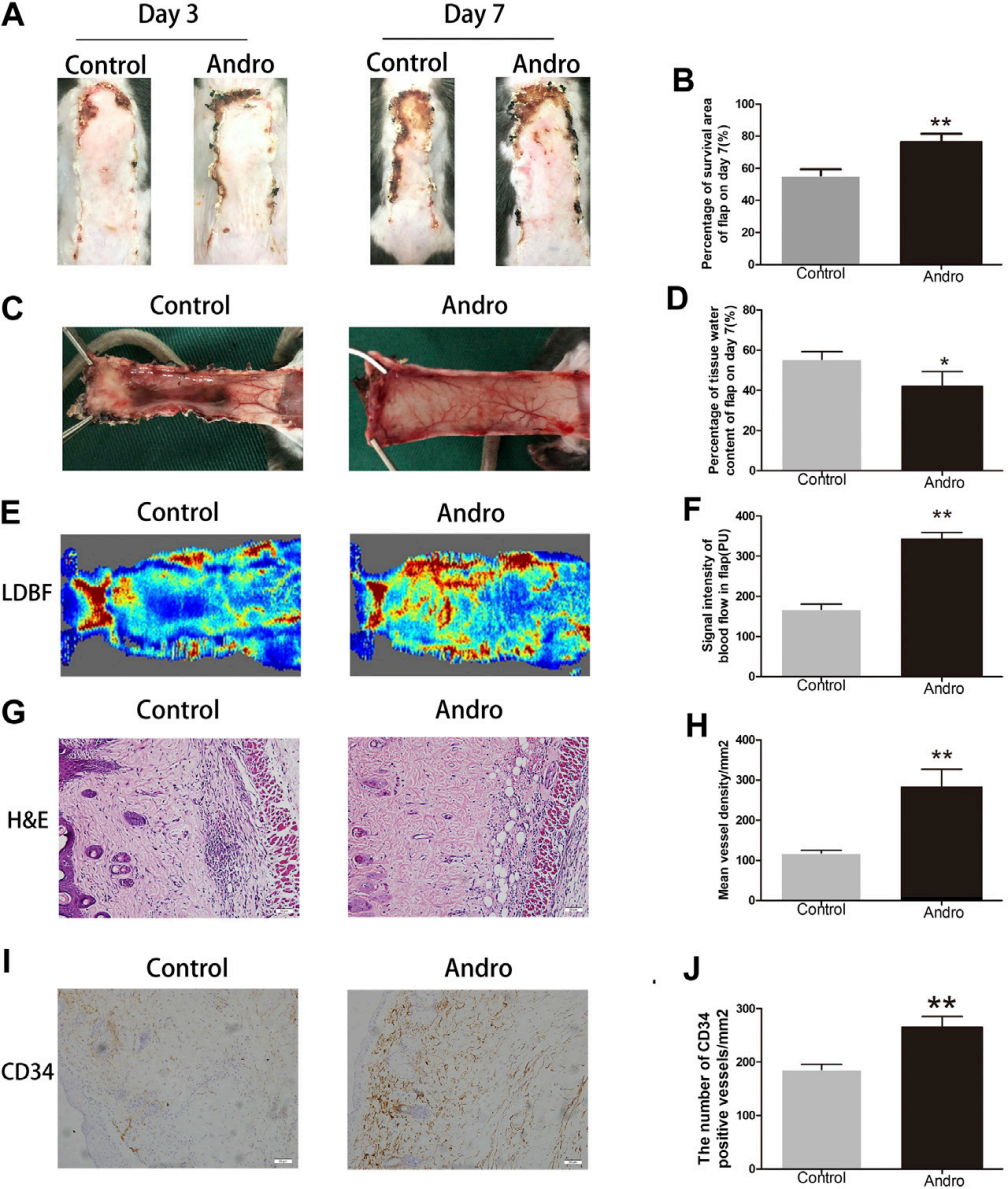
Andro enhances the random-pattern skin flap survival **(A)** The digital images of survival/necrosis area in both groups after the operation (on the third day and seventh day). **(B)** The histogram of survival area percentage on postoperative day seventh. **(C)** The image of tissue edema and necrosis in the Control and Andro group on day seventh of the post-surgery. **(D)** The histogram reveals the percentage of water content in tissues. **(E)** The blood supply and vascular flow in both groups. **(F)** The histogram shows the signal intensities of the blood flow in flaps. **(G)** The H&E staining between two groups showing the vessels (original magnification ×200; scan bar, 50 μm). **(H)** The histogram depicts the MVD percentage. **(I)** The IHC 0f CD34 to mark vessels in vascular endothelial cells in the skin flap (original magnification ×200; scale bar, 50 µm). **(J)** The histogram depicts the CD34-positive vessel density percentage. The obtained data were presented as means ± SEM. Significance: *p-value < 0.05 and **p-value < 0.01, vs. control group (n = 6 per group).

After establishing the random-pattern skin flap mice model, the flaps distal parts slowly began to appear pale and were swollen. On postoperative day 3, no effective variation was found in the viability of flaps among two groups, as depicted in Figure 2A. On day seventh after surgery, the necrosis in the dorsum of the skin was larger and darker compared with the groups on day 3. The survival area of the flap in the group exposed to Andro was effectively improved in comparison with the control group (74.32 ± 4.50 and 53.68 ± 4.41%, respectively; *p* = 0.006; Figure 2B). According to the qualitative analysis, the control group flaps were puffy compared to the Andro group (Figure 2C). The water content of flaps represents the tissue edema, we found that the amount of water in the control was elevated in comparison with the Andro group (55.97 ± 8.97 and 38.42 ± 4.23%, respectively; *p* = 0.01; Figure 2D). The results implied that the edema and retention of water in tissues were lightened via the Andro exposure. Furthermore, the microvascular network was reconstructed and determined via LDBF, as depicted in Figure 2E. After analysis of data, the blood flow signal intensity was considerably higher in the Andro group in comparison with the control group, as shown in Figure 2F (320.03 ± 38.75°PU and 187.69 ± 35.62°PU, respectively; *p* = 0.005). Eosin and Hematoxylin Staining were used for the identification of vessel density (Figure 2G), and microvessels were effectively elevated in the Andro group relative to control, as depicted in Figure 2H (240.68 ± 42.38 and 110.35 ± 10.58/mm^2^, respectively; *p* = 0.008). Likewise, CD34 immunohistochemistry (CD34 IHC) was considerably elevated in the Andro group relative to the control group (242.68 ± 32.52 and 181.53 ± 12.63/mm^2^, respectively; *p* = 0.004; Figures 2I,J). Taken together, the underlined results showed that Andro has a significant contribution to flap viability.

### Andro Upregulates Angiogenesis in the Skin Flaps

**FIGURE 3:**
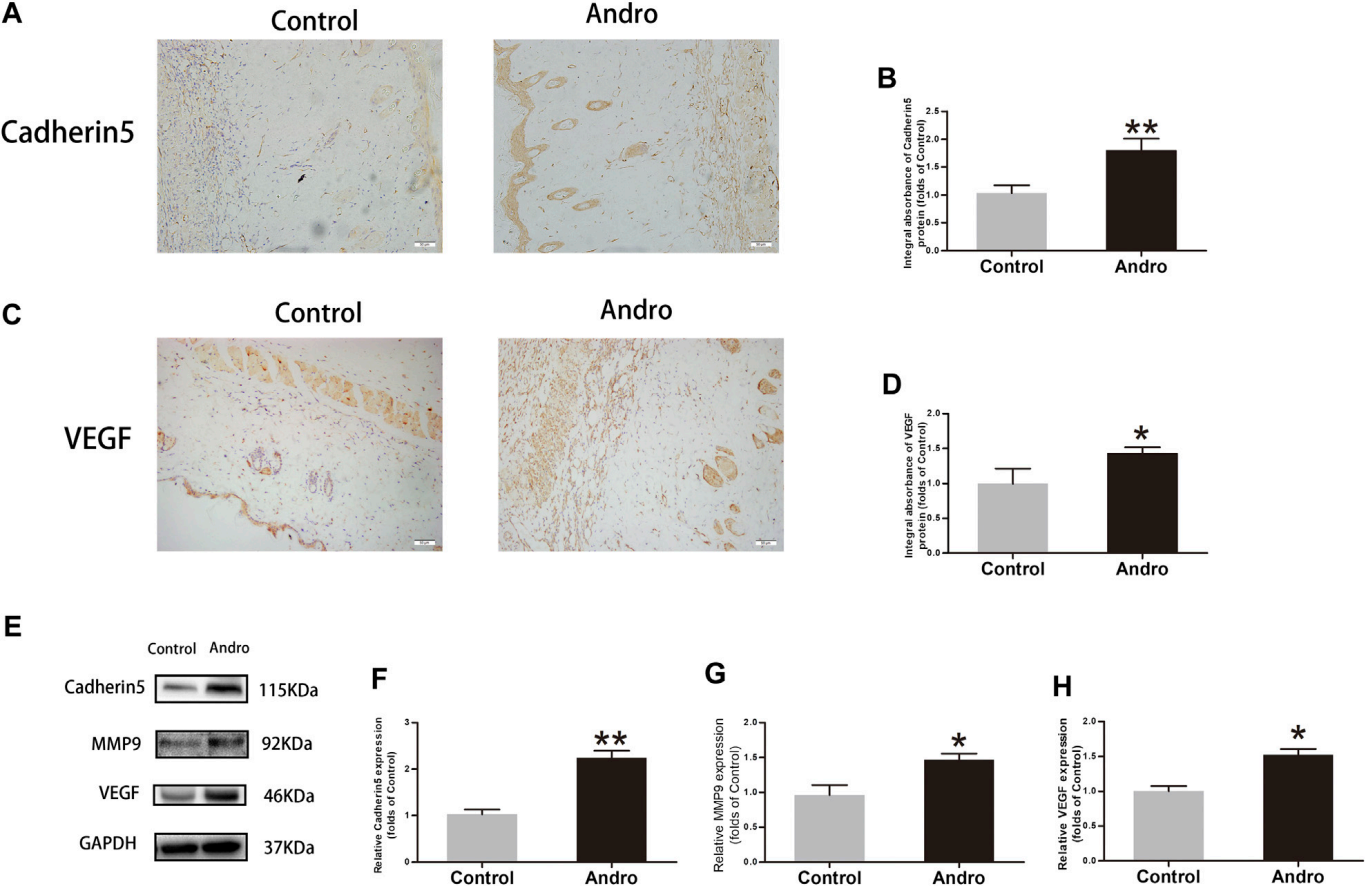
The upregulation of angiogenesis in the skin flaps via Andro **(A,C)** IHC of Cadherin5 and VEGF in both groups of ischemic skin flaps (original magnification, ×200; scan bar, 50 μm). **(B,D)** The total absorbance of Cadherin5 and VEGF in IHC. **(E)** The results of immunoblotting i.e., the expressions of cadherin 5, MMP9, VEGF in the control as well as Andro groups. **(F–H)** The quantification of Cadherin5, MMP9, and VEGF expressions in the flaps by measuring their optical densities. The obtained data were presented as means ± SEM. Significance: *p-value < 0.05 and **p-value < 0.01, vs. control group (n = 6 per group).

To investigate whether Andro upregulates angiogenesis in the ischemia area of flaps, a series of expression markers of angiogenesis was measured by using IHC and western blotting. The obtained results indicated that Cadherin5 expressed normally in stromal cells and vessels, elevated considerably in the Andro group relative to the control group, as indicated in Figures 3A,B (*p* = 0.004; Figure 3B). Similarly, in IHC, the VEGF integral absorbance was determined in stromal cells and vessels and its expression was elevated via Andro treatment in immunoblotting (*p* = 0.02, Figure 3D; *p* = 0.03, Figure 3H). Moreover, the expression level of MMP9 was also increased via Andro (*p* = 0.01, Figure 3G). The above results revealed that angiogenesis was upregulated via Andro which is a significant factor of skin flap viability through stimulating the expression level of the VEGF, Cadherin5, and MMP9.

### Andro Suppresses the Apoptotic Process in the Skin Flaps

**FIGURE 4:**
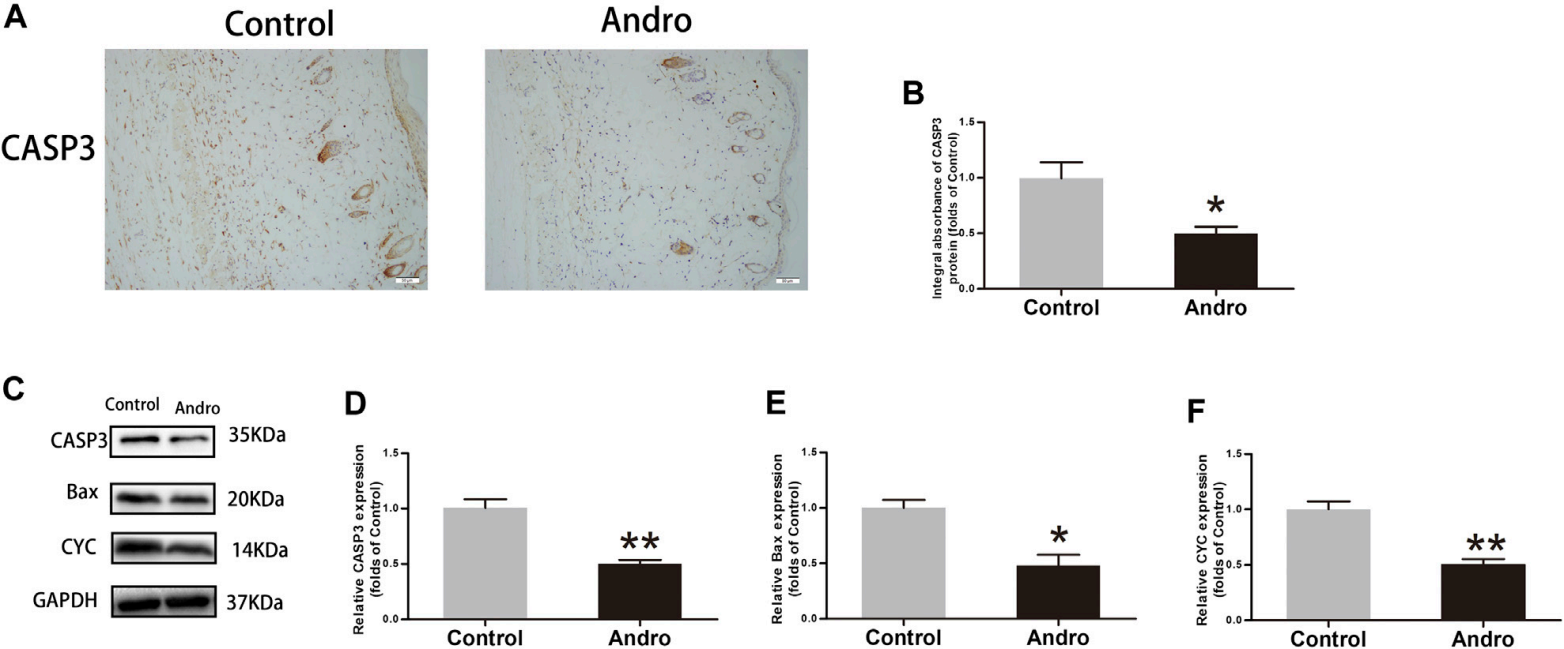
Apoptosis inhibition in the skin flaps via Andro **(A)** The CASP3 expression evaluated via IHC in flaps (original magnification ×200; scale bar, 50 µm). **(B)** The histogram representing the CASP3 total absorbance in IHC. **(C)** The immunoblotting of CASP3, Bax, and CYC expressions in each group. **(D–F)** The quantification of CASP3, Bax, and CYC expressions in the flaps by measuring their optical densities. The obtained data were presented as means ± SEM. Significance: *p-value < 0.05 and **p-value < 0.01, vs. control group (n = 6 per group).

To evaluate whether Andro suppresses the apoptotic process, IHC and immunoblotting were used to investigate the expressions of proteins correlated with the apoptotic process. The CASP3 expression level was found to be decreased in the Control group, as depicted in Figure 4A. The CAPS3 integral absorbance was decreased in the Andro group relative to the control group (*p* = 0.02, Figure 4B). Furthermore, immunoblotting was employed to evaluate the expression level of CYC, CASP3, and Bax in the flaps, as indicated in Figure 4C. It was revealed that the expression level of Bax, CYC, and CASP3 in the Andro group was lowered compared to the control group (*p* = 0.007, Figure 4D; *p* = 0.03, Figure 4E; *p* = 0.004, Figure 4F). Collectively, the underlined findings demonstrate that the active treatment of Andro on the skin flaps survival owing to the inhibition of apoptosis.

### Andro Alleviates Oxidative Stress in the Skin Flaps

**FIGURE 5:**
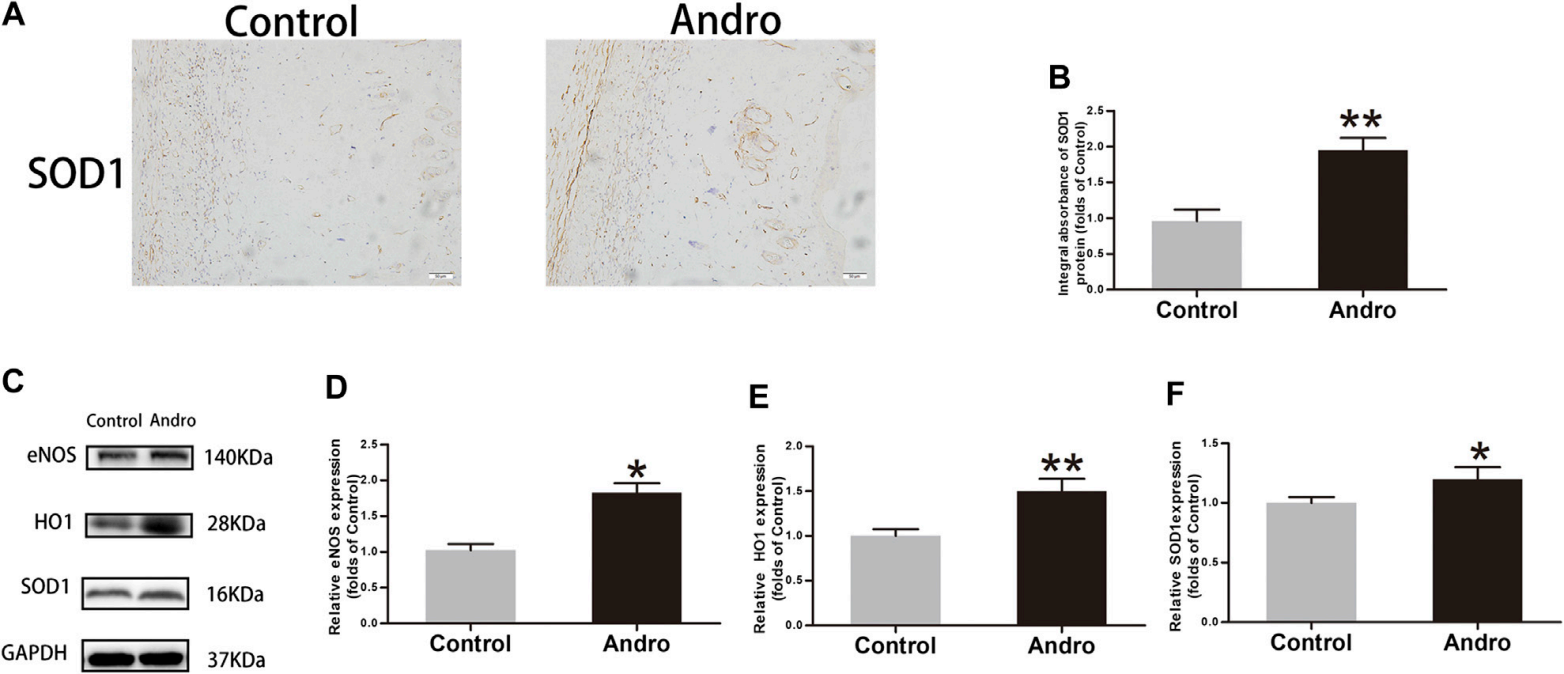
The alleviation of oxidative stress in the skin flaps via Andro **(A)** The **e**xpression of SOD1 evaluated via IHC in flaps (original magnification ×200; scale bar, 50 µm). **(B)** The histogram representing the total SOD1 absorbance in IHC. **(C)** The immunoblotting of SOD1, HO1and eNOS expressions in each group. **(D–F)** The quantification of SOD1, HO1and eNOS expressions in the flaps by measuring their optical densities. The obtained data were presented as means ± SEM. Significance: *p-value < 0.05 and **p-value < 0.01, vs. control group (n = 6 per group).

Oxidative stress has a significant contribution to the necrosis of skin flaps. To reveal whether Andro alleviates oxidative stress, IHC was employed for the identification of SOD1 level in the skin flaps, as depicted in Figure 5A. The obtained results indicated that Andro treatment effectively increased the SOD1 level and its integral absorbance (*p* = 0.008; Figure 5B). The results of immunoblotting indicated the overexpression of proteins i.e., HO1, eNOS, and SOD1 in the Andro group comparative to the control group, as depicted in Figures 5C–F. (*p* = 0.04, *p* = 0.006, *p* = 0.03, respectively; Figures 5D–F). The underlined proteins have been closely correlated with oxidative stress. The obtained results suggested that Andro enhanced the survival of the flap and it may be due to the decreased level of oxidative stress by Andro in the ischemic area of flaps.

### Andro Enhances Autophagy in the Skin Flaps

**FIGURE 6:**
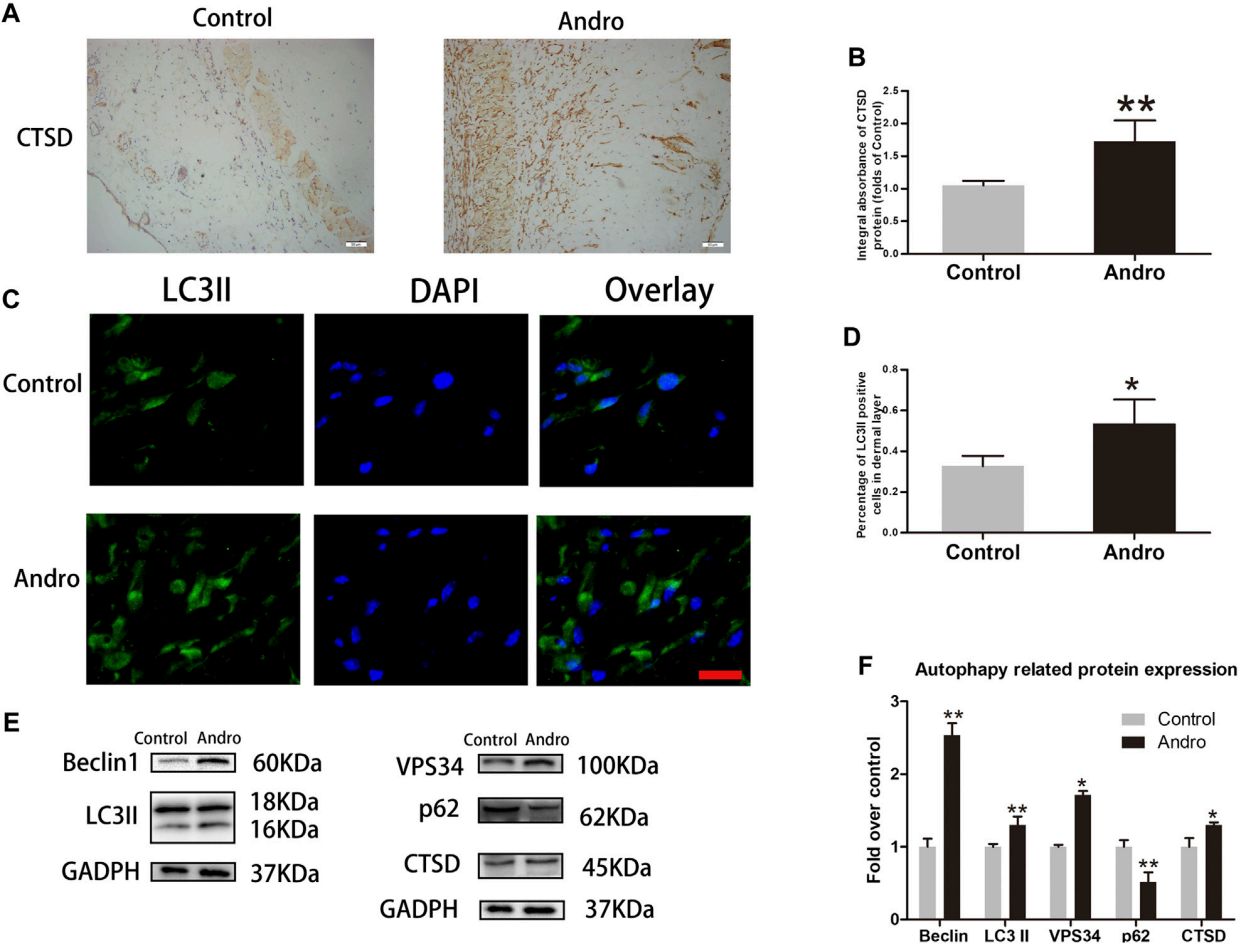
The enhancement of autophagy in the skin flaps via Andro **(A) T**he **e**xpression of CTSD evaluated via IHC in flaps (original magnification ×200; scale bar, 50 µm). **(B) T**he histogram representing the total CTSD absorbance in IHC. **(C)** The immunofluorescence results of LC3II in the ischemic skin flap (scan bar, 50 μm). **(D)** The histogram depicts the percentage of the LC3II-positive cells. **(E)** The immunoblotting of Beclin 1, LC3II, CTSD, VPS34, and p62 expressions in each group. **(F)** The quantification of Beclin 1, LC3II, CTSD, VPS34, and p62 expressions in the flaps by measuring their optical densities. The obtained data were presented as means ± SEM. Significance: *p-value < 0.05 and **p-value < 0.01, vs. control group (n = 6 per group).

It has been indicated that Andro effectively contributes to apoptosis, oxidative stress, and angiogenesis. Herein, we speculated that Andro may attribute partially to the regulatory mechanism of autophagy. Consequently, the expression level of autophagy-associated proteins was tested to ensure whether Andro has a positive influence on the ischemic area of flaps. Proteins i.e., LC3II, VPS34, and Beclin1 are essential components of autophagosomes while CTSD is a lysosomal marker, and p62 is an indicator of autophagic flux. The elevated expression of CTSD was found in the dermal layer of the Andro group than the control, as depicted in Figure 6A. Likewise, the CTSD integral absorbance indicates considerably elevated expression levels of CTSD than the control (*p* = 0.004; Figure 6B). In immunofluorescence, the autophagosomes and nuclei were labeled with LC3II punctate dots and DAPI with the emission of green and blue colors, respectively. A large number of LC3II-positive cells were identified in the dermis of the Andro group relative to the control group, as depicted in Figures 6C,D (*p* = 0.032; Figure 6D). Furthermore, western blotting results demonstrated that in the Andro group the elevated expression levels of VPS34, Beclin1, CTSD, and LC3II were found in the flap tissues, while the decreased expression level of p62 was also observed in the underlined tissues of the Andro group (*p* = 0.042, *p* = 0.008, *p* = 0.048, *p* = 0.006, *p* = 0.009, respectively; Figure 6F). These results suggested that Andro enhanced autophagy in the flaps ischemic area.

### 3MA Reverses the Influence of Andro on Random-Pattern Skin Flap Viability

**FIGURE 7:**
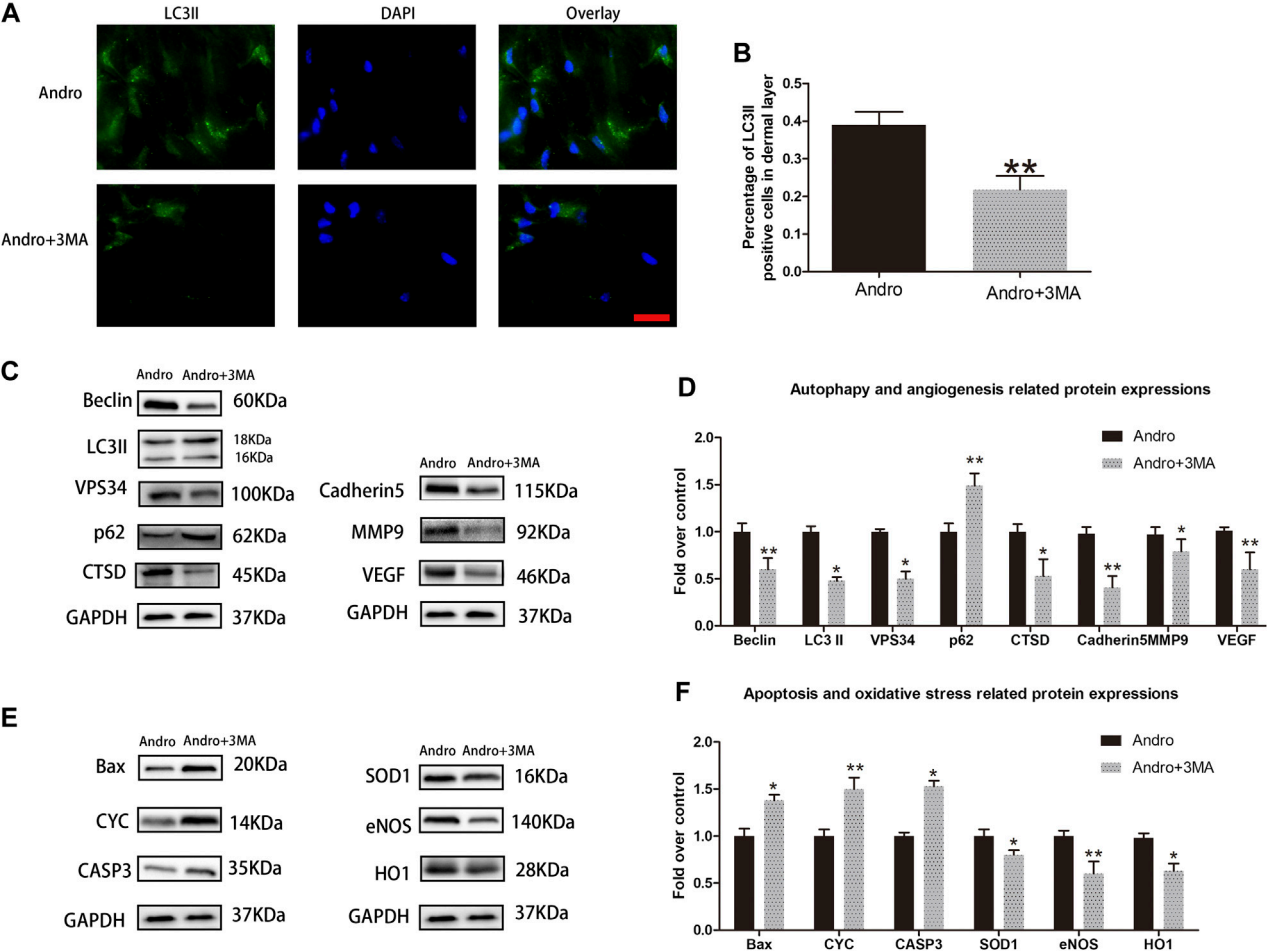
3MA reversed the effects of Andro on random-pattern skin flap viability **(A)** The immunofluorescence data of LC3II in the ischemic skin flap (scan bar, 50 μm). **(B) T**he histogram representing the percentage of the LC3II-positive cells. **(C,E)** The immunoblotting of the autophagy associated proteins VPS34, p62, LC3II, Beclin 1, and CTSD; angiogenesis associated proteins VEGF, cadherin5, and MMP9; the oxidative stress-related proteins *i.e.,* SOD1, HO1, and eNOS and the apoptosis-associated proteins *i.e.,* Bax, CYC, and CASP3. **(D,F)** The quantification of autophagy-, angiogenesis, and oxidative stress-related proteins by measuring their optical densities, as previously mentioned. The obtained data were presented as means ± SEM. Significance: *p-value < 0.05 and **p-value < 0.01, vs. control group (n = 6 per group).

To assess whether Andro enhances the survival of random pattern skin flap through stimulating autophagy, we combined Andro with 3MA (a potent inhibitor of autophagy), and then the results were analyzed. Initially, we confirmed that 3MA co-administered with Andro inhibit autophagy. The immunofluorescence and immunoblotting assays were conducted to identify the autophagy markers. A considerable elevation in the percentage of LC3II positive cells was observed in the dermal layer of the Andro group in comparison with the Andro+3MA group, as depicted in Figures 6A,B. Similar results were also obtained from the immunoblotting assay.The 3MA co-administered with Andro significantly reduced the level of LC3II, VPS34, Beclin 1, and CTSD (*p* = 0.027, *p* = 0.032, *p* = 0.008, and *p* = 0.041, respectively; Figures 7C,D), while elevated the expression of p62, as compared with the Andro treatment alone (*p* = 0.008, Figure 7D). The apoptosis-related proteins, such as CASP3, Bax, and CYC were overexpressed in the Andro group in comparison with the Andro+3MA group (*p* = 0.024, *p* = 0.018, and *p* = 0.007, respectively; Figures 7E,F). The oxidative stress-related protein i.e., eNOS, SOD1, and HO1 were underexpressed in the Andro+3MA group relative to the Andro group (*p* = 0.005, *p* = 0.033, and *p* = 0.042, respectively; Figures 7E,F). The above results showed that 3MA co-administered with Andro inhibited the random-pattern skin flap viability.

### Suppression Autophagy Reverses the Effect of Andro on Flap Vitality

**FIGURE 8:**
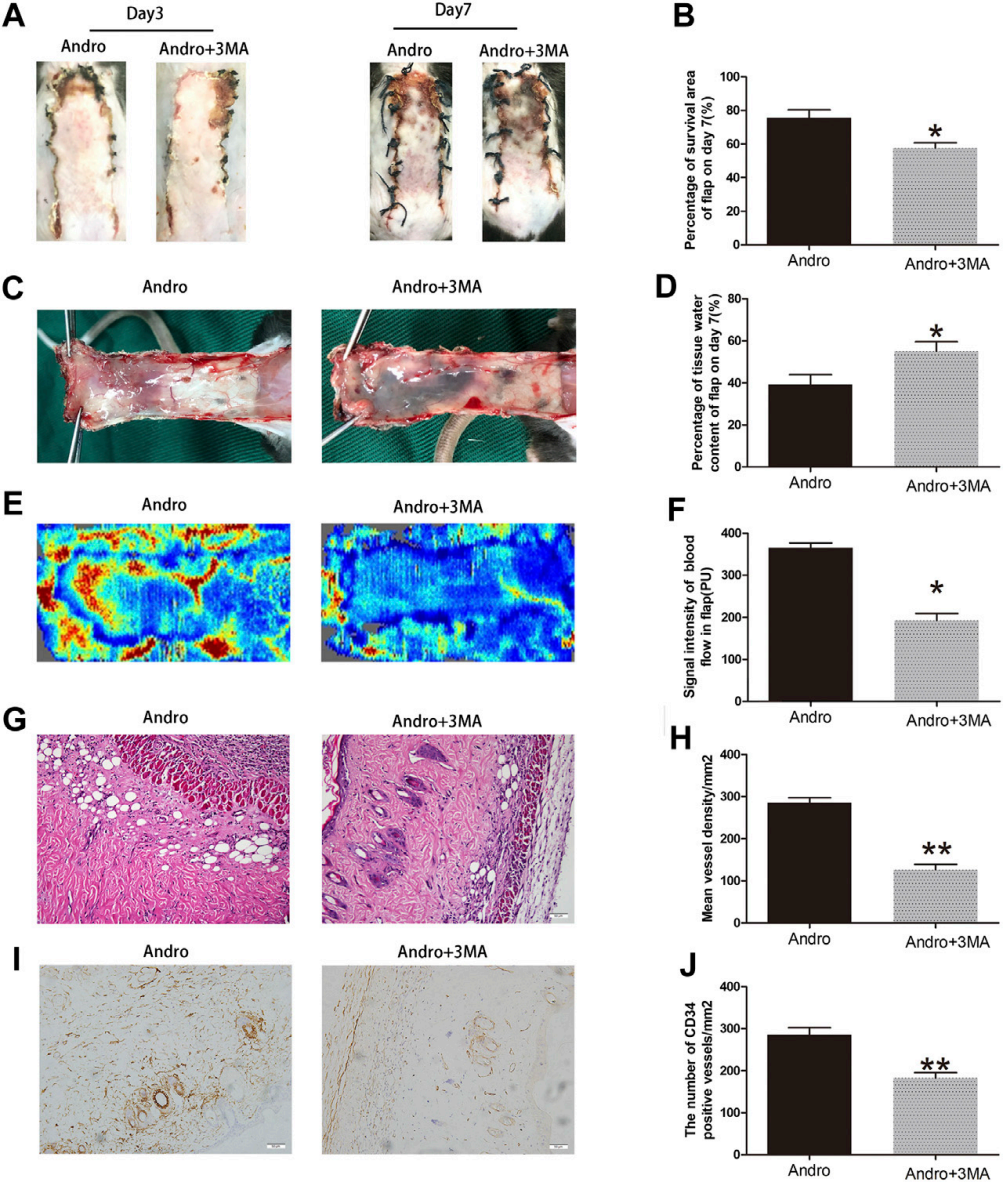
Suppression of autophagy reversed the effects of Andro on flap vitality **(A)** The digital images of survival/necrosis area in Andro and Andro+ 3-methyladenine (3MA) groups after the operation (POD3 and POD7). **(B)** The histogram of survival area percentage on postoperative day seventh. **(C)** The image of tissue edema and necrosis in the Andro group and Andro+3MA group on the seventh day after surgery. **(D)** The histogram reveals the percentage of water content in tissue. **(E)** The blood supply and vascular flow in both groups. **(F)** The histogram shows the signal intensities of the blood flow in flaps. **(G)** The staining of H&E (original magnification ×200; scan bar, 50 μm). **(H)** The histogram depicts the MVD percentage. **(I) T**he IHC 0f CD34 to mark vessels in vascular endothelial cells in the skin flap (original magnification ×200; scale bar, 50 µm). **(J) T**he histogram depicts the percentage of CD34-positive vessel density. The obtained data were presented as means ± SEM. Si/gnificance: *p-value < 0.05 and **p-value < 0.01, vs. control group (n = 6 per group).

To evaluate whether 3MA co-administered with the Andro impact flap viability, the random-pattern skin flaps were performed to compare the appearance of skin flaps at different postoperative points. The digital pictures showed that flap viability in the Andro+3MA group was considerably decreased in comparison with the Andro treatment on postoperative day 7, as depicted in Figures 8A,B (76.53 ± 4.56 and 57.34 ± 6.38%, respectively; *p* = 0.031; Figure 8B). Likewise, the distal parts of the flap were swollen and bruised in the Andro+3MA group (Figure 8C), leading to a statistical difference in the water content of flap tissues (37.59 ± 4.23 and 57.43 ± 6.28%, respectively; *p* = 0.021; Figure 8D). LDBF results indicated higher blood flow signal intensity in the Andro group, as depicted in Figure 8E, and after statistical analysis, the results were statistically different (369.15 ± 37.45 and 182.45 ± 27.42°PU, respectively; *p* = 0.021; Figure 8F). H&E staining was employed to evaluate the number of microvascular networks in the dermis of tissue (Figure 8G). The Andro+3MA group revealed that the vascular density mean was considerably reduced in comparison with the Andro group (282.37 ± 32.78 and 112.58 ± 23.89/mm^2^, respectively; *p* = 0.007; Figure 8H). In conclusion, as indicated in Figure 8I, the IHC of CD34 positive blood vessels were significantly decreased in the 3MA co-administered with Andro (276.47 ± 39.43 and 163.49 ± 28.26/mm^2^; *p* = 0.008; Figure 8J).

### Andro Attenuates Apoptosis by Regulating the PI3K/Akt Signaling Pathway

**FIGURE 9:**
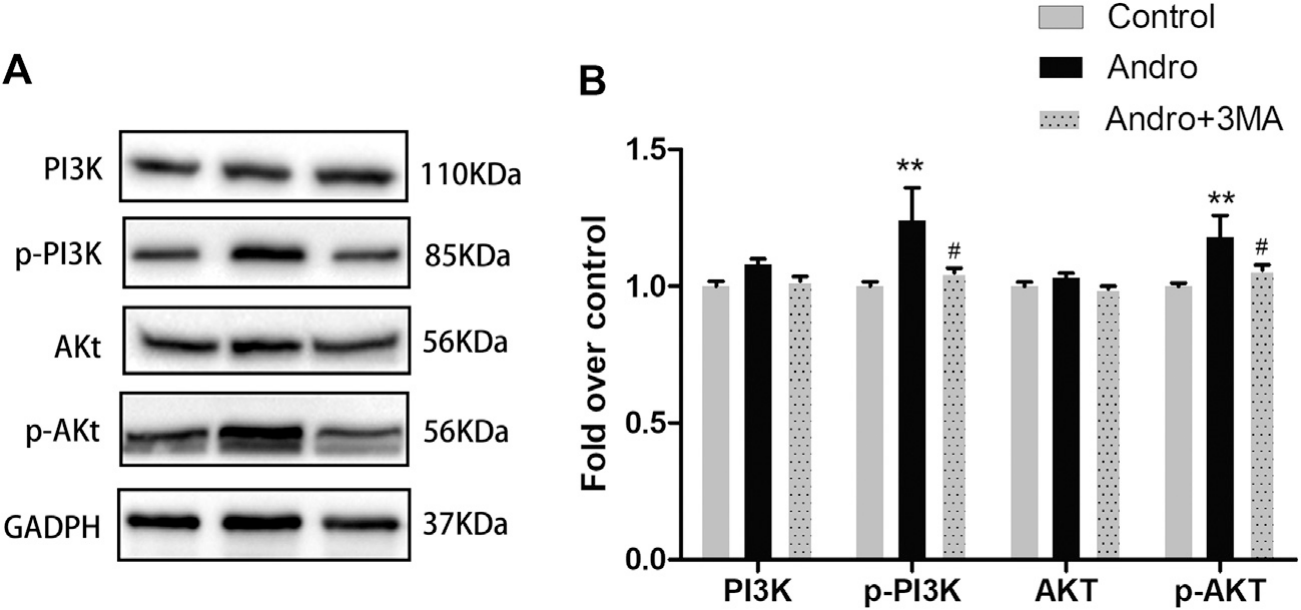
Andro Attenuates Apoptosis by Regulating the PI3K/Akt Signaling Pathway **(A)** The immunoblotting expressions of PI3K, p-PI3K, Akt, and *p*-Akt in the control, Andro, and Andro+3MA groups. **(B)** The quantification of PI3K,p-PI3K,Akt, and *p*-Akt expressions in the each group by measuring their optical densities. *p < 0.05 and **p < 0.01, vs. control group; #*p* < 0.05, ##*p* < 0.01, vs. Andro group (n = 6 per group).

Previous research have revealed that Andro protects cells from apoptosis through activation PI3K/Akt signaling pathway (Duan et al., 2019). Meanwhile, 3MA is not only an autophagy inhibitor, but also a selective PI3K inhibitor. Therefore, we applied the westen blotting to detect the expression levels of PI3K/Akt pathway related proteins to verify whether Andro-mediated autophagy is involved in this pathway. Our results showed that the expression of phosphorylated PI3K and Akt in the Andro group was higher than Andro+3MA group and the control group (Figures 9A,B). There are no significantly difference in the expression levels of PI3K and Akt in three groups (Figures 9A,B). Our results confirmed that Andro can reduce apoptosis by regulating the PI3K/Akt pathway in random pattern skin flap, which explains why the anti-apoptotic effect of Andro is weakened after inhibiting autophagy.

## Discussion

Andrographis paniculata Nees is a medicinal plant that is widely used to treat respiratory infections and inflammation in Asia for centuries (Li et al., 2020). Andrographolide (Andro) is one of the most important bicyclic diterpene lactones that has been extracted from the leaves of *A. paniculata*. Andro possesses a remarkable inhibitory activity against inflammation, cancer, and viral infections (Gupta et al., 2020). Current studies have revealed that it improves microcirculation and autophagy, while inhibits oxidative stress and cellular apoptotic process (Yang et al., 2014; Geng et al., 2019; Farooqi et al., 2020; Li et al., 2020). However, there are few studies on Andro-induced autophagy especially in flap models. We established that Andro increased survival of random-pattern skin flaps and inhibited Andro-induced autophagy can reverse positive effects of Andro on random pattern flap survival.

Andro can improve myocardial ischemia and reduce reperfusion injury (Xie et al., 2020). To evaluate the role of Andro in angiogenesis, we conducted H&E staining and CD34-positive IHC staining of vascular cells, and the blood vessel density in the flap tissues was found to be elevated in the Andro group comparing to the control group. Furthermore, the LDBF analysis indicated that the intensity of blood flow was elevated in the Andro group. The underlined results revealed that the treatment with Andro elevates the viability of skin flaps via enhancing blood supply and angiogenesis. The new blood vessels are produced from preexisting blood vessels, while the mitosis, proliferation, germination, and migration of endothelial cells also participate in the generation of new blood vessels (Ahrens et al., 2003; Longchamp et al., 2018; Mărginean et al., 2019). MMP9 enhances the remodeling of vascular tissue through the disconnection of intercellular bridges between fully developed vascular cells (Li et al., 2018). VEGF plays a significant role in different processes of angiogenesis (mainly in mitosis of vascular cells), while Cadherin five enhances the generation of new blood vessels along with its maturation (Li et al., 2018). In this study, we identified that the underlined indicators are closely correlated with angiogenesis. Based on IHC and immunoblotting results, in the stromal cells and dermal blood vessels, the expression level of MMP9, VEGF, and cadherin five were elevated by Andro treatment. In summary, we concluded that Andro is an accelerator of angiogenesis via the upregulation of cadherin 5, VEGF, and MMP9.

IRI plays a important role in flap necrosis (Chen et al., 2018). In the injury and repair mechanism, the restricted blood flow in the tissues has resumed, and as result, blood carries oxygen molecules. The superoxide anions are formed by the reaction of these oxygen molecules which initiate lipid peroxidation and disrupt the plasma membrane. The underlined consequences cause necrosis and generate MDA (Yao et al., 2018). In this view, the underlined process, the enzymes linked with antioxidant activity have a key role in the prevention of oxidative stress (Kumar et al., 2016). SOD, eNOS, and Heme oxygenase one have a significant antioxidant activity (Sun et al., 2018). In this study, the analysis of IHC and immunoblotting have indicated that the expression of eNOS, SOD1, and HO1 was elevated in the Andro group. Collectively, the underlined results revealed that Andro plays an effective role against IRI and oxidative stress. Some studies have also reported that Andro contributes significantly to the suppression of the apoptotic process (Xiang et al., 2020; Khan et al., 2021). For instance, in human osteosarcoma, the apoptotic process has been caused by Andro via ROS/JNK cascade (Wang et al., 2020). Apoptosis is a key determinant of cells and tissues’ survival (Xu et al., 2020). So, we determined whether Andro induced the downregulation of the apoptotic process in the dermis of the random-pattern skin flaps and accelerated flap survival. The mitochondrial-mediated apoptosis can be triggered by different kinds of cellular stresses and tends to release CYC from mitochondria into the cytosol (Fuchs and Steller, 2015). A pro-apoptotic protein called Bax has a key contribution to the regulation of CYC release (Kitamatsu et al., 2021). Hence, we evaluated the expression of CYC, Bax and CASP3 to identify the level of programmed cell death. In this study, it has also been indicated that the expression level of CASP3, CYC and Bax are effectively suppressed via Andro in dermal cells of ischemic flap tissues which reveals that Andro suppress apoptotic process in animal random flaps.

Autophagy is a conservative self-degradation process and maintains the stability of the intracellular environment (Wang et al., 2014). Autophagy is a cellular adaptation in response to critical environmental conditions (Parzych and Klionsky, 2014). It removes denatured and damaged proteins and nonfunctional organelles to prevent cellular damage. To further explore the role of Andro in the elevation of skin flap viability, autophagy was analyzed in the skin flap model (Itakura and Mizushima, 2010). Autophagy is a catabolic process that degrades damaged biomolecules and organelles by lysosomal cascades through generating autophagosome, fused with the lysosome, and results in the degradation of the autophagic substrate (Parzych and Klionsky, 2014). In the current work, an increased level of autophagy markers has been noticed in the Andro group than the control group. We investigated the underlined indicators: Beclin1, LC3II, VPS34 (indicators of autophagosomes); CTSD (an indicator of autolysosomes) and p62 (an indicator of autophagic degradation). After Andro treatment, the percentage of LC3II-positive cells was elevated in the dermis in comparison with the control group. The total absorbance of CTSD indicated that the elevated expression level of Cathepsin D was found in the Andro group than the control group. In the current study, western blotting results also indicated that the expression level of Beclin 1, LC3II and VPS34 was considerably elevated in the Andro group, indicating that additional autophagosomes were formed in the random flaps. In addition, overexpression of CTSD and underexpression of p62 were detected in the ischemic flap tissues of the Andro group, suggesting higher autophagy flux. The underlined results revealed that Andro increases the autophagy of random-pattern skin flaps.

A high level of autophagy is not all good for cells. As the activation of autophagy enhanced the survival of cells, but in several diseases, including acute myocardial infarction, autophagy excessive activation may contribute to the apoptotic process (Zou et al., 2019). Hence, in the existing study, the Andro-mediated autophagy was determined in the random flaps. 3-Methyladenine is a common autophagy inhibitor (Wang et al., 2016; Miller et al., 2010). This study revealed that 3MA co-administered with Andro inhibit autophagy, thus inhibited the random-pattern skin flap viability. Herein, we determined that 3MA treatment effectively decreased the expression of Cadherin 5, MMP9, and VEGF. So, we hypothesized that Andro accelerates flap angiogenesis via activating autophagy. The mitochondrial apoptosis which has been caused via deficiency of nutrients can be inhibited via activation of autophagy (Miyazaki et al., 2015). Our results also indicated that the expression level of CYC, Bax, and CASP3 were reduced post 3MA treatment which showed that the Andro anti-apoptotic effects have been caused via autophagy activation. Autophagy is a catabolic process that degrades damaged and dysfunctional ubiquitinated proteins of mitochondria. Activation of the autophagic system can remove the oxidizing components of the cell in oxidative stress response and contribute considerably in adaptation to oxidative stress (Dutta et al., 2013). 3MA decreased the expression of HO1, SOD1, and eNOS. Hence, Andro lowers the oxidative stress of random flaps by inducing autophagy.

Interaction between apoptosis and Andro-induced autophagy mentioned previously prompted us to further study the mechanism by which Andro’s anti-apoptotic effect was weakened after co-administration with 3MA. 3MA is a selective PI3K inhibitor. The activation of PI3K/Akt signaling pathway can inhibit caspase activation during apoptosis and up-regulate the activity of Bcl-2 family proteins (New et al., 2007). In addition, Andro can activate PI3K/Akt signaling pathway to protect cells from apoptosis (Kumar et al., 2015). In this study, we detected PI3K/Akt signaling pathway related proteins. Subsequently, our findings showed that Andro-induced autophagy can reduce the apoptosis of random pattern skin flap by activating the PI3K/Akt signaling pathway, and 3MA reverses Andro’s anti-apoptotic effect by inhibiting PI3K phosphorylation.

In general, our research still has some limitations, which need to further exploration in future research. Firstly, although it has been determined that Andro-induced autophagy is beneficial to flap survival, the specific pathways that promote autophagy should still be studied. Secondly, due to the lack of *in vitro* experiments, it is unclear whether there are more factors involved in Andro-induced autophagy. However, Andro-induced autophagy improves flap survival and provides a solid foundation for future research.

## Conclusion

In summary, our present work supports that Andro can elevate the skin flap viability by enhancing angiogenesis, attenuating cellular apoptotic process, and decreasing the level of oxidative stress through enhancing autophagy.

## Data Availability

The raw data supporting the conclusions of this article will be made available by the authors, without undue reservation.
